# The treatment and outcomes of early-stage epithelial ovarian cancer: have we made any progress?

**DOI:** 10.1038/sj.bjc.6604299

**Published:** 2008-03-18

**Authors:** J K Chan, K Fuh, J Y Shin, M K Cheung, C B Powell, L-m Chen, D S Kapp, K Osann

**Affiliations:** 1 Division of Gynecologic Oncology, Department of Obstetrics, Gynecology and Reproductive Sciences, UCSF Helen Diller Family Comprehensive Cancer Center, University of California, San Francisco School of Medicine, 1600 Divisadero Street, Box 1702, San Francisco, CA 94143, USA; 2 Division of Gynecologic Oncology, Department of Obstetrics and Gynecology, Stanford Cancer Center, Stanford University School of Medicine, 875 Blake Wilbur Drive, MC 5827, Stanford, CA 94305, USA; 3 Division of Radiation Therapy, Department of Radiation Oncology, Stanford Cancer Center, Stanford University School of Medicine, 875 Blake Wilbur Drive, MC 5827, Stanford, CA 94305, USA; 4 Division of Hematology and Oncology, Department of Medicine, Chao Family Comprehensive Cancer Center, University of California, Irvine Medical Center, 101 City Drive, Orange, CA 92868, USA

**Keywords:** epithelial ovarian cancer, early stage, progress

## Abstract

The objective of this study is to determine the progress and trends in the treatment and survival of women with early-stage (I–II) epithelial ovarian cancer. Data were obtained from the SEER database between 1988 and 2001. Kaplan–Meier and Cox regressions methods were employed for statistical analyses. Of the 8372 patients, the median age was 57 years (range: 12–99 years). A total of 6152 patients (73.4%) presented with stage I and 2220 (26.5%) with stage II disease. Over the periods 1988–1992, 1993–1997, and 1998–2001, 3-year disease-specific survivals increased from 86.1 to 87.2 to 88.8% (*P*=0.076). The number of patients that underwent lymphadenectomy has increased significantly from 26.2 to 38.7 to 54.2% over the study period (*P*<0.001). Of those patients who underwent staging procedures with lymphadenectomy, there was no improvement in survival over the three study periods (from 93.2 to 93.5 to 93.1%; *P*=0.978). On multivariate analysis, younger age, nonclear cell histology, earlier stage, lower grade, surgery, and lymphadenectomy were significant independent prognostic factors for improved survival. After adjusting for surgical staging with lymphadenectomy, the year of diagnosis was no longer an important prognostic factor. In conclusion, the use of lymphadenectomy during surgery for early-stage ovarian cancer has doubled over the last 14 years. The marginal improvement in survival demonstrated over time is potentially attributed to the increased use of staging procedures with lymphadenectomy.

Ovarian cancer is the fifth leading cause of cancer death in women and the second most common gynaecologic cancer in the United States (Jemal *et al*, 2006). In 2007, an estimated 22 430 new epithelial ovarian cancers were diagnosed in the United States and approximately one-third had FIGO (International Federation of Obstetrics and Gynecology) stage I and II disease with a survival rate ranging from 70 to 90% (Heintz *et al*, 2006; Jemal *et al*, 2006). Although the survival of early-stage disease is significantly higher than those with advanced cancers, approximately 20–30% of patients with early-stage cancers will succumb to their disease (Nguyen *et al*, 1993; Hoskins *et al*, 1994; Kosary, 1994; Averette *et al*, 1995; McGuire *et al*, 1996; Heintz *et al*, 2006).

Prior reports have shown that age, stage, cell type, tumour grade, large volume ascites, and dense adhesions are important clinical and pathological prognostic factors (Dembo *et al*, 1990; Sevelda *et al*, 1990; Young *et al*, 1990; Finn *et al*, 1992; Bertelsen *et al*, 1993; Vergote *et al*, 1993; Sjovall *et al*, 1994; Ahmed *et al*, 1996; Holschneider and Berek, 2000; Trope *et al*, 2000). Recently, Chan *et al* (2008) reported on patients with high-risk early-stage patients defined as stage I, grade 3; stage IC; stage II; or clear cell epithelial ovarian cancer after adjuvant therapy from two Gynecologic Oncology Group studies. These authors also found that age, stage, grade, and cytology are important prognostic factors in these patients (Chan *et al*, 2008).

Despite the fact that advanced stage disease is associated with a poorer survival, a recent study showed that these women had a significant improvement in 5-year survival from 25.4 to 29.4% over time. However, this study was not able to demonstrate a statistically significantly benefit in survival in women with early-stage cancers (Chan *et al*, 2006). The objective of this study was to evaluate the demographic, clinicopathologic, treatment, and survival trends of patients with early-stage epithelial ovarian cancer, and to determine the prognostic factors responsible for specific survival trends.

## MATERIALS AND METHODS

Demographic, clinicopathologic, treatment, and survival information of women diagnosed with stage I–II epithelial ovarian cancer during the period from 1988 through 2001 were identified from the Surveillance, Epidemiology and End Results (SEER) (2005) of the National Cancer Institute. The SEER program is an epidemiologic surveillance system sponsored by the National Cancer Institute consisting of population-based tumour registries that routinely collect information on all incidents of cancer occurring in persons residing in SEER areas of the US. Patient demographic data, cancer data (such as histology, stage, and grade), diagnosis date, surgical treatment, and radiation therapy recommended and/or provided within 4 months of diagnosis, follow-up of vital status, and cause of death, if applicable is recorded. The SEER data do not contain information about comorbidity or treatments received beyond the 4 months following diagnosis. As of 2002, the SEER areas include the states of Connecticut, Hawaii, Iowa, New Mexico, and Utah, as well as the metropolitan areas of Detroit, San Francisco – Oakland, Los Angeles, San Jose, Atlanta, and Seattle – Puget Sound. The latest expansion includes the addition of areas in four states – Kentucky, Louisiana, New Jersey, and the remainder of California. The SEER program encompasses 25% of the US population in varied demographic areas.

In all, 8372 women were diagnosed with early-stage ovarian carcinoma from 1988 to 2001 and were divided into three time intervals: 1988–1992, 1993–1997, and 1998–2001. Factors including age at diagnosis, race, marital status, stage, tumour histology, grade of disease, type of surgery, and disease-specific survival were extracted. Race was classified into four groups, including Caucasian, African American, Asian, and Hispanic.

*χ*^2^ tests were performed to analyse trends in the study cohort over the three time periods, 1988–1992, 1993–1997, and 1998–2001. Kaplan–Meier analyses for 3-year survival were performed on the 1988–1992, 1993–1997, and 1998–2001 time intervals. The outcome of interest was death from ovarian cancer as determined by the underlying cause of death on the death certificate. Thus, time to death was censored in women who died from causes other than ovarian cancer and who were alive at last follow-up. Cox proportional hazards were used for multivariable analyses. Two-tailed tests at *P*-values less than 0.05 were considered significant. All data were analysed using SPSS 15.0 (SPSS, Chicago, IL, USA) and SAS (version 6.12; SAS Inc., Cary, NC, USA).

## RESULTS

In all, 8372 women were diagnosed with early-stage ovarian carcinoma from 1988 to 2001. Table 1 shows demographic and clinical characteristics of these women. Median age was 57 years with 66.6% 50 years of age or older. Across the three time intervals, 1988–1992, 1993–1997, and 1998–2001, there was an increase in the proportion of Hispanics and Asians diagnosed with early-stage cancers (*P*<0.001). More specifically, the proportion of Caucasians diagnosed with ovarian cancer decreased from 84.6 to 76.8 to 74.4%. Conversely, the proportion of Asians increased from 4.7 to 7.8 to 8.9% and the proportion of Hispanics increased from 4.8 to 7.6 to 8.5%. The number of patients that underwent lymphadenectomy has increased significantly from 26.2 to 38.7 to 54.2% over the study period (*P*<0.001). In all, 73.4% were categorised as stage I and 26.5% were categorised as stage II disease. There was no significant change in the proportion of cases, which were stage I or II over the three time periods (*P*=0.253). Histologically, 26.4% were serous, 26.6% endometrioid, 19.1% mucinous, 11.2% clear cell, and 16.6% were other epithelial cell types. An increase in the proportion of serous and endometrioid histology was seen in the latter time period, whereas there was a decrease in the mucinous subtype (*P*<0.001). A total of 20.3% of women had grade 1, 25.8% grade 2, and 26.5% had grade 3 disease. There was an increase in the percentage of grade 3 disease throughout the years (from 22.5 to 27.4 to 29.3%; *P*=0.010).

**Figure 1:**
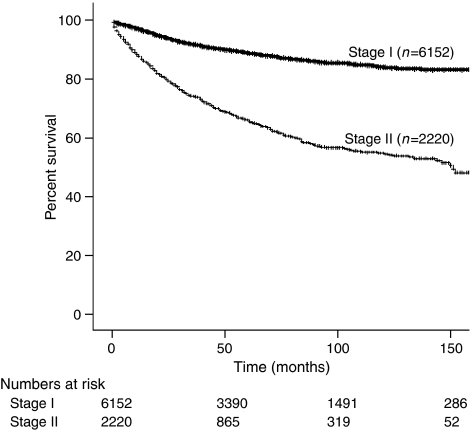
Kaplan–Meier disease-specific survival by stage (*P*<0.001).

**Figure 2:**
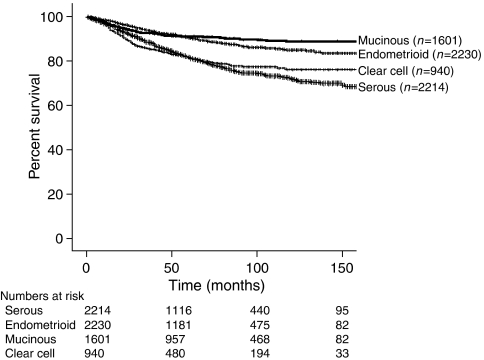
Kaplan–Meier disease-specific survival by histology (*P*<0.001).

For women who were younger than 50 years of age, 3-year survival was 93.1% compared with 84.2% for those who were 50 years old and older (*P*<0.001). Three-year disease-specific survival among Hispanics, Asians, Caucasians, and African Americans also differed (88.8 *vs* 89.4 *vs* 87.1 *vs* 84.5%) (*P*=0.005). Stage I patients had a significantly improved survival at 91.8% compared with 74.2% in those with stage II disease (*P*<0.001) (Figure 1). Comparing the four major epithelial histologic cell types, endometrioid has a statistically significant increase in 3-year disease-specific survival compared with the other histologies as seen in Table 2 and Figure 2 (*P*=0.015). Grade 1 tumours were found to have a higher 3-year disease-specific survival at 96.4% compared with grades 2 and 3, at 92.4 and 82.0%, respectively (*P*<0.001).

**Figure 3:**
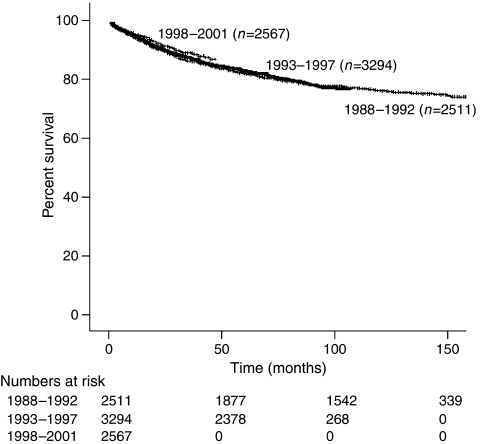
Kaplan–Meier disease-specific survival by year (*P*=0.076).

Over the 3 time intervals from 1988 to 1992, 1993 to 1997, and 1998 to 2001, women diagnosed with early-stage epithelial ovarian carcinoma had a marginal improvement in survival from 86.1 to 87.2 to 88.8% (*P*=0.076) (Figure 3). During these time periods, the 3-year survival was estimated based on age, race, surgery, lymphadenectomy, stage, histologic cell type, and grade of disease for each of the time periods (Table 2). Of note, there was a survival benefit in the women ⩾50 years (*P*=0.048), endometrioid histology (*P*=0.015), and grade 3 disease (*P*<0.001) over the three time periods. Although the use of lymphadenectomy has increased over time, of those patients who underwent staging procedures with lymphadenectomy, there was no improvement in survival over the three periods (from 93.2 to 93.5 to 93.1%; *P*=0.978). A lack of significant improvement in disease-specific survival over the three time periods studied was also seen when separate analyses were performed for stage I and II patients with or without lymphadenectomy (Table 2).

In our multivariate model, year of diagnosis, younger age, surgery, earlier stage, nonclear cell histology, and lower grade were significant independent prognostic factors for improved survival (Table 3A). However, after adjusting for surgical staging with lymphadenectomy, the year of diagnosis had a marginal significance (HR=0.99, CI: 0.97–1.00; *P*=0.098) (Table 3B). The two multivariate models demonstrate the relationship between year of diagnosis and effect of surgical staging with lymphadenectomy.

## DISCUSSION

Prior studies on early-stage ovarian cancer patients have consisted of a heterogeneous group with respect to risk of recurrence and survival. These studies have shown that patients with early-stage disease have overall survival ranging from 60 to 100% (Nguyen *et al*, 1993; Kosary, 1994; Averette *et al*, 1995; Partridge *et al*, 1996; Creasman *et al*, 2003; Jemal *et al*, 2006). Previous reports on ovarian cancer survival estimates were based on patients diagnosed many years ago with outdated estimates (Young *et al*, 1990; Yancik, 1993). In addition, many of these reports were limited by the lack of International Federation of Gynecology and Obstetrics (FIGO) staging and histologic information (Brenner, 2002; Engel *et al*, 2002; Barnholtz-Sloan *et al*, 2003). This current report is one of the largest population-based studies that consist exclusively of early-stage epithelial ovarian cancer patients with histologic and surgical information. In over 8000 women diagnosed with early ovarian cancer, we only showed a marginal improvement in survival over the last 14 years. Thus, we determined the factors that are responsible for these findings based on demographic and clinicopathologic predictors.

Despite the significant progress in treatment of advanced ovarian cancers over the last 10 years, we have not improved the survival of young patients. The authors recognise that approximately 10% of young women with ovarian cancer had germ cell tumours with survival rates that have reached over 90%. As such, it may be difficult to detect a survival benefit in these young patients given that these patients have excellent survivals from their germ cell cancers. However, the lack of survival improvement in young women may only be partially explained by the higher proportion of germ cell tumours compared with the older cohorts. In this study, we showed that women <50-years old with early-stage epithelial ovarian cancer did not have an improvement in survival over time (Table 2). Some studies have found that a significant number of these young patients with poor prognostic ovarian cancers do not undergo adjuvant chemotherapy. Cress *et al* (2003) studied 2150 women with ovarian cancer and found that approximately 20% of patients younger than 55 years with stage IC and II ovarian cancer did not receive chemotherapy. However, the likelihood of receiving chemotherapy was significantly increased if a gynaecologic oncologist was involved in the patient's care (Chan *et al*, 2007a). Furthermore, Asians had a superior 5-year survival at 57.2% compared with African Americans (45.5%) and Caucasians (46.6%) (*P*<0.001). A subanalysis revealed that the Asian patients in our study presented at a younger age, earlier stage, and lower grade of disease than their counterparts – all factors that contribute to the better survival in this racial group.

Despite the better survival in early-stage ovarian cancer compared with advanced stage cancer, there has been no significant improvement in survival over the years for early ovarian cancer. In fact, stage II ovarian cancer continues to carry a 3-year survival of 70–77% with no improvement in survival over the years. Several studies have shown that stage of disease within those with early-stage cancers is an important prognostic factor (Schildkraut *et al*, 2000; Pectasides *et al*, 2007). Given the poor prognosis of stage II patients compared with stage I patients, many investigators have advocated for the inclusions of stage II patients into clinical trials for advanced (stages III–IV) cancers.

In this study, clear cell tumours have a worse survival compared with the other histological subtypes. Previous analyses have shown that clear cell tumours carry a worse prognosis compared with other epithelial malignancies adjusted for stage of disease (Vergote *et al*, 1993). However, other studies have found no significant difference between clear cell and other epithelial subtypes (Pettersson, 1988). In our multivariate analyses, clear cell histology, stage II disease, and poorly differentiated tumours all were independent factors for poor prognosis. Although it is reassuring that survival has improved with poorly differentiated tumours over the years, these findings are not evident in stage II disease and clear cell histology.

Since 1988, the International Federation of Gynecology and Obstetrics published guidelines for surgical staging for ovarian cancer that included pelvic and para-aortic lymph node dissection or lymphadenectomy. Over time, the use of lymphadenectomy during ovarian cancer surgery has increased. Up to 30% of patients were found, on prior studies, to be upstaged from early-stage ovarian cancer during the restaging procedure (Young *et al*, 1983; Helewa *et al*, 1986; Soper *et al*, 1992). It is also possible that this association of lymphadenectomy and better survival is attributed to more appropriate treatment due to more accurate staging. In our first multivariate model, we found that year of diagnosis was an independent prognostic factor for improved survival over time (Table 3A). However, after adjusting for the increased use of lymphadenectomy over time, the improvement in outcome was no longer evident (Table 3B). Thus, it is likely that the survival improvement associated with lymphadenectomy over time is due to an increase in proportion of true early-stage patients after a thorough staging procedure, and subsequent removal of inaccurately staged patients with true stage IIIC disease. Similarly, other reports have described a possible association between lymphadenectomy and better survival in early nonclear cell epithelial ovarian cancer (Chan *et al*, 2007b). In addition, this association was attributed to accurate staging leading to appropriate treatment and, possibly, the removal of micrometastatic disease within the node, which would have been considered negative on pathological analyses.

This study is limited by its retrospective design. Even though the SEER database has value in determining treatment and survival trends, treatment claims must be used with caution. For example, there is a lack of information on the specialty of surgeon and detailed information on the types and cycles of chemotherapy. One inherent advantage of the SEER database is the ability to generalise these results in a population comparable to that of the United States. This is also the largest study, to date, investigating the survival trends and prognostic factors in early-stage epithelial ovarian cancer.

The use of lymphadenectomy during surgery for early-stage ovarian cancer has doubled over the last 14 years. The marginal improvement in survival demonstrated over time is potentially attributed to the increased use of staging procedures with lymphadenectomy.

## Figures and Tables

**Table 1:** Demographic and clinicopathologic characteristics

	**Total**	**1988–1992**	**1993–1997**	**1998–2001**	***P*-value**
Overall	8372	2511	3294	2567	
					
*Age at diagnosis (years)*					
Median (range)	57 (12–99)	58 (12–99)	57 (15–99)	55 (14–97)	
Age <50	2799 (33.4%)	836 (33.2%)	1110 (33.7%)	853 (33.2%)	0.917
Age ⩾50	5573 (66.6%)	1675 (66.8%)	2184 (66.3%)	1714 (66.8%)	
					
*Race*					
Caucasian	6564 (78.4%)	2125 (84.6%)	2530 (76.8%)	1909 (74.4%)	<0.001
Hispanic	587 (7.0%)	120 (4.8%)	250 (7.6%)	217 (8.5%)	
African American	401 (4.8%)	105 (4.2%)	174 (5.3%)	122 (4.7%)	
Asian	605 (7.2%)	118 (4.7%)	257 (7.8%)	230 (8.9%)	
Other	215 (2.6%)	43 (1.7%)	83 (2.5%)	89 (3.5%)	
					
*Surgery*					
Yes	7945 (94.9%)	2406 (95.8%)	3102 (94.2%)	2437 (94.9%)	0.018
No	427 (5.1%)	105 (4.2%)	192 (5.8%)	130 (5.1%)	
					
*Lymphadenectomy*					
Yes	3327 (39.7%)	659 (26.2%)	1276 (38.7%)	1392 (54.2%)	<0.001
No	4360 (52.1%)	1648 (65.6%)	1713 (52.0%)	999 (38.9%)	
Unknown	685 (8.2%)	204 (8.1%)	305 (9.3%)	176 (6.9%)	
					
*Stage*					
Stage I	6152 (73.4%)	1853 (73.8%)	2443 (74.2%)	1856 (72.3%)	0.253
Lymphadenectomy	2506 (29.9%)	510 (20.3%)	964 (29.3%)	1032 (40.2%)	<0.001
No lymphadenectomy	3120 (37.3%)	1188 (47.3%)	1237 (37.6%)	695 (27.1%)	
Stage II	2220 (26.5%)	658 (26.2%)	851 (25.8%)	711 (27.7%)	
Lymphadenectomy	821 (9.8%)	149 (5.9%)	312 (9.5%)	360 (14.0%)	<0.001
No lymphadenectomy	1240 (14.8%)	460 (18.3%)	476 (14.4%)	304 (11.8%)	
					
*Histology*					
Serous	2214 (26.4%)	671 (26.7%)	847 (25.7%)	696 (27.1%)	<0.001
Endometrioid	2230 (26.6%)	574 (22.9%)	875 (26.6%)	781 (30.4%)	
Mucinous	1601 (19.1%)	552 (22.0%)	641 (19.5%)	408 (15.9%)	
Clear cell	940 (11.2%)	256 (10.2%)	380 (11.5%)	304 (11.8%)	
Other	1387 (16.6%)	458 (18.2%)	551 (16.7%)	378 (14.7%)	
					
*Grade*					
Grade 1	1703 (20.3%)	474 (18.9%)	717 (21.8%)	512 (19.9%)	0.010
Grade 2	2163 (25.8%)	635 (25.3%)	834 (25.3%)	694 (27.0%)	
Grade 3	2219 (26.5%)	566 (22.5%)	902 (27.4%)	751 (29.3%)	
Unknown	2287 (27.3%)	836 (33.3%)	841 (25.5%)	610 (23.8%)	

**Table 2:** Three-year disease-specific survival

	**Total (%)**	**1988–1992 (%)**	**1993–1997 (%)**	**1998–2001 (%)**	**Log-rank**
Overall	87.2 (±0.4)	86.1 (±0.7)	87.2 (±0.6)	88.8 (±0.8)	*P*=0.076
					
*Age at diagnosis (years)*					*P*<0.001Δ
<50	93.1 (±0.5)	93.8 (±0.8)	92.2 (±0.8)	94.0 (±1.1)	*P*=0.259*
⩾50	84.2 (±0.5)	82.2 (±1.0)	84.5 (±0.8)	86.3 (±1.1)	*P*=0.048*
					
*Race*					*P*=0.005Δ
Caucasian	87.1 (±0.4)	86.2 (±0.8)	86.7 (±0.7)	88.2 (±1.0)	*P*=0.374*
Hispanic	88.8 (±1.5)	90.3 (±2.8)	86.7 (±2.2)	91.1 (±2.8)	*P*=0.395*
African American	84.5 (±2.0)	80.9 (±4.0)	86.3 (±2.7)	85.1 (±4.2)	*P*=0.213*
Asian	89.4 (±1.4)	84.7 (±3.4)	90.7 (±1.9)	91.0 (±2.6)	*P*=0.495*
					
*Surgery*					*P*<0.001Δ
Yes	90.1 (±0.4)	88.4 (±0.7)	90.7 (±0.5)	91.5 (±0.8)	*P*=0.678*
No	24.8 (±2.6)	22.2 (±4.9)	22.3 (±3.5)	34.1 (±5.2)	*P*=0.022*
					
*Lymphadenectomy*					*P*<0.001Δ
Yes	93.3 (±0.5)	93.2 (±1.0)	93.5 (±0.7)	93.1 (±0.9)	*P*=0.978*
No	82.0 (±0.6)	82.8 (±1.0)	81.2 (±1.0)	82.0 (±1.6)	*P*=0.211*
					
*Stage*					*P*<0.001Δ
Stage I	91.8 (±0.4)	91.4 (±0.7)	91.5 (±0.6)	93.4 (±0.8)	*P*=0.202*
					*P*<0.001Δ
Lymphadenectomy	95.2 (±0.5)	95.0 (±1.0)	94.7 (±0.7)	96.3 (±0.8)	*P*=0.468*
No lymphadenectomy	89.0 (±0.6)	90.0 (±0.9)	88.4 (±0.9)	88.6 (±1.6)	*P*=0.295*
Stage II	74.2 (±1.0)	70.7 (±1.8)	74.5 (±1.5)	77.3 (±2.1)	*P*=0.057*
					*P*<0.001Δ
Lymphadenectomy	87.4 (±1.3)	87.0 (±2.8)	89.5 (±1.8)	84.3 (±2.7)	*P*=0.425*
No lymphadenectomy	63.4 (±1.5)	63.2 (±2.4)	62.1 (±2.3)	67.0 (±3.5)	*P*=0.410*
					
*Histology*					*P*<0.001Δ
Serous	88.4 (±0.7)	86.6 (±1.3)	89.4 (±1.1)	88.9 (±1.7)	*P*=0.412*
Endometrioid	93.8 (±0.6)	92.1 (±1.1)	93.5 (±0.8)	96.7 (±0.8)	*P*=0.015*
Mucinous	92.5 (±0.7)	93.1 (±1.1)	92.9 (±1.0)	90.2 (±1.9)	*P*=0.460*
Clear cell	85.8 (±1.2)	84.4 (±2.3)	84.9 (±1.9)	87.2 (±3.0)	*P*=0.863*
					
*Grade*					*P*<0.001Δ
1	96.4 (±0.5)	96.5 (±0.9)	96.1 (±0.7)	96.6 (±1.1)	*P*=0.875*
2	92.4 (±0.6)	92.2 (±1.1)	92.1 (±0.9)	93.3 (±1.2)	*P*=0.676*
3	82.0 (±0.9)	75.9 (±1.9)	83.3 (±1.3)	86.7 (±1.7)	*P*<0.001*

Δ *P*-value represents differences in survival of patients <50 *vs* ⩾50 years, Caucasian *vs* Hispanics *vs* African American *vs* Asian, surgery *vs* no surgery, lymphadenectomy *vs* no lymphadenectomy, Stage I *vs* Stage II, serous *vs* endometrioid *vs* mucinous *vs* clear cell histologies, and grades 1 *vs* 2 *vs* 3.

* *P*-value represents differences in survival over time based on demographic and clinicopathologic prognostic factors.

**Table 3:** Multivariate analysis

**Prognostic factor**	**Hazard ratio**	**95% confidence interval**	***P*-value**
*(A) Including lymphadenectomy*			
Year of diagnosis^a^	0.98	0.96–0.99	0.004
Age at diagnosis^a^	1.03	1.02–1.03	<0.001
Surgery^b^	0.17	0.15–0.20	<0.001
Stage^c^	2.45	2.19–2.75	<0.001
Histology^d^	1.30	1.20–1.42	<0.001
Grade^e^	1.57	1.42–1.73	<0.001
			
*(B) Excluding lymphadenectomy*			
Year of diagnosis^a^	0.99	0.97–1.00	0.098
Age at diagnosis^a^	1.03	1.02–1.03	<0.001
Surgery^b^	0.19	0.16–0.23	<0.001
Lymphadenectomy^b^	0.68	0.59–0.78	<0.001
Stage^c^	2.48	2.22–2.78	<0.001
Histology^d^	1.28	1.17–1.39	<0.001
Grade^e^	1.56	1.43–1.73	<0.001

a Continuous (1988–2001).

b No *vs* yes.

c I *vs* II.

d Endometrioid or mucinous *vs* serous or clear cell *vs* other.

e 1 *vs* 2 *vs* 3+unknown.

## References

[bib1] Ahmed FY, Wiltshaw E, A’Hern RP, Nicol B, Shepherd J, Blake P, Fisher C, Gore ME (1996) Natural history and prognosis of untreated stage I epithelial ovarian carcinoma. J Clin Oncol 14: 2968–2975 8918494 10.1200/JCO.1996.14.11.2968

[bib2] Averette HE, Janicek MF, Menck HR (1995) The National Cancer Data Base report on ovarian cancer. American College of Surgeons Commission on Cancer and the American Cancer Society. Cancer 76: 1096–1103 8625213 10.1002/1097-0142(19950915)76:6<1096::aid-cncr2820760626>3.0.co;2-4

[bib3] Barnholtz-Sloan JS, Schwartz AG, Qureshi F, Jacques S, Malone J, Munkarah AR (2003) Ovarian cancer: changes in patterns at diagnosis and relative survival over the last three decades. Am J Obstet Gynecol 189: 1120–1127 14586365 10.1067/s0002-9378(03)00579-9

[bib4] Bertelsen K, Holund B, Andersen JE, Nielsen K, Stroyer I, Ladehoff P (1993) Prognostic factors and adjuvant treatment in early epithelial ovarian cancer. Int J Gynecol Cancer 3: 211–218 11578348 10.1046/j.1525-1438.1993.03040211.x

[bib5] Brenner H (2002) Long-term survival rates of cancer patients achieved by the end of the 20th century: a period analysis. Lancet 360: 1131–1135 12387961 10.1016/S0140-6736(02)11199-8

[bib6] Chan JK, Cheung MK, Husain A, Teng NN, West D, Whittemore AS, Berek JS, Osann K (2006) Patterns and progress in ovarian cancer over 14 years. Obstet Gynecol 108: 521–528 16946210 10.1097/01.AOG.0000231680.58221.a7

[bib7] Chan JK, Kapp DS, Shin JY, Husain A, Teng NN, Berek JS, Osann K, Leiserowitz GS, Cress RD, O’Malley C (2007a) Influence of the gynecologic oncologist on the survival of ovarian cancer patients. Obstet Gynecol 109(6): 1342–1350 17540806 10.1097/01.AOG.0000265207.27755.28

[bib8] Chan JK, Munro EG, Cheung MK, Husain A, Teng NN, Berek JS, Osann K (2007b) Association of lymphadenectomy and survival in stage I ovarian cancer patients. Obstet Gynecol 109: 12–19 17197582 10.1097/01.AOG.0000249610.95885.ef

[bib9] Chan JK, Tian C, Monk BJ, Herzog T, Kapp DS, Bell J, Young RC (2008) Prognostic factors for high-risk early-stage epithelial ovarian cancer: a Gynecologic Oncology Group Study. Cancer (in press) 10.1002/cncr.23390 18348296

[bib10] Creasman WT, Odicino F, Maisonneuve P, Beller U, Benedet JL, Heintz AP, Ngan HY, Pecorelli S (2003) Carcinoma of the corpus uteri. Int J Gynaecol Obstet 83(Suppl 1): 79–118 14763170 10.1016/s0020-7292(03)90116-0

[bib11] Cress RD, O’Malley CD, Leiserowitz GS, Campleman SL (2003) Patterns of chemotherapy use for women with ovarian cancer: a population-based study. J Clin Oncol 21: 1530–1535 12697877 10.1200/JCO.2003.08.065

[bib12] Dembo AJ, Davy M, Stenwig AE, Berle EJ, Bush RS, Kjorstad K (1990) Prognostic factors in patients with stage I epithelial ovarian cancer. Obstet Gynecol 75: 263–273 2300355

[bib13] Engel J, Eckel R, Schubert-Fritschle G, Kerr J, Kuhn W, Diebold J, Kimmig R, Rehbock J, Holzel D (2002) Moderate progress for ovarian cancer in the last 20 years: prolongation of survival, but no improvement in the cure rate. Eur J Cancer 38: 2435–2445 12460789 10.1016/s0959-8049(02)00495-1

[bib14] Finn CB, Luesley DM, Buxton EJ, Blackledge GR, Kelly K, Dunn JA, Wilson S (1992) Is stage I epithelial ovarian cancer overtreated both surgically and systemically? Results of a five-year cancer registry review. Br J Obstet Gynaecol 99: 54–58 1547174 10.1111/j.1471-0528.1992.tb14393.x

[bib15] Heintz AP, Odicino F, Maisonneuve P, Benedet JL, Creasman WT, Ngan HY, Pecorelli S, Beller U (2006) Carcinoma of the ovary. Int J Gynaecol Obstet 95(S1): S161–S192 10.1016/S0020-7292(06)60033-7 29644669

[bib16] Helewa ME, Krepart GV, Lotocki R (1986) Staging laparotomy in early epithelial ovarian carcinoma. Am J Obstet Gynecol 154: 282–286 3946515 10.1016/0002-9378(86)90656-3

[bib17] Holschneider CH, Berek JS (2000) Ovarian cancer: epidemiology, biology, and prognostic factors. Semin Surg Oncol 19: 3–10 10883018 10.1002/1098-2388(200007/08)19:1<3::aid-ssu2>3.0.co;2-s

[bib18] Hoskins PJ, Swenerton KD, Manji M, Wong F, O’Reilly SE, McMurtrie EJ, Le N, Acker B, Le Richer J (1994) ‘Moderate-risk’ ovarian cancer (stage I, grade 2; stage II, grade 1 or 2) treated with cisplatin chemotherapy (single agent or combination) and pelvi-abdominal irradiation. Int J Gynecol Cancer 4: 272–278 11578417 10.1046/j.1525-1438.1994.04040272.x

[bib19] Jemal A, Siegel R, Ward E, Murray T, Xu J, Smigal C, Thun MJ (2006) Cancer statistics, 2006. CA Cancer J Clin 56: 106–130 16514137 10.3322/canjclin.56.2.106

[bib20] Kosary CL (1994) FIGO stage, histology, histologic grade, age and race as prognostic factors in determining survival for cancers of the female gynecological system: an analysis of 1973–1987 SEER cases of cancers of the endometrium, cervix, ovary, vulva, and vagina. Semin Surg Oncol 10: 31–46 8115784 10.1002/ssu.2980100107

[bib21] McGuire WP, Hoskins WJ, Brady MF, Kucera PR, Partridge EE, Look KY, Clarke-Pearson DL, Davidson M (1996) Cyclophosphamide and cisplatin *versus* paclitaxel and cisplatin: a phase III randomized trial in patients with suboptimal stage III/IV ovarian cancer (from the Gynecologic Oncology Group). Semin Oncol 23: 40–47 8941409

[bib22] Nguyen HN, Averette HE, Hoskins W, Sevin BU, Penalver M, Steren A (1993) National survey of ovarian carcinoma. VI. Critical assessment of current International Federation of Gynecology and Obstetrics staging system. Cancer 72: 3007–3011 8221569 10.1002/1097-0142(19931115)72:10<3007::aid-cncr2820721024>3.0.co;2-n

[bib23] Partridge EE, Phillips JL, Menck HR (1996) The National Cancer Data Base report on ovarian cancer treatment in United States hospitals. Cancer 78: 2236–2246 8918420 10.1002/(sici)1097-0142(19961115)78:10<2236::aid-cncr28>3.0.co;2-z

[bib24] Pectasides D, Fountzilas G, Aravantinos G, Bamias A, Kalofonos HP, Skarlos D, Briasoulis E, Konstantara A, Economopoulos T, Dimopoulos MA (2007) Epithelial ovarian carcinoma in younger *vs* older women: is age an independent prognostic factor? The Hellenic Oncology Cooperative Group experience. Int J Gynecol Cancer 17: 1003–1010 17367314 10.1111/j.1525-1438.2007.00912.x

[bib25] Pettersson F (1988) International Federation of Gynecology and Obstetrics annual report on the results of treatment in gynecologic cancer, Vol. 20, p 110. Stockholm, Sweden: Panorama Press AB

[bib26] Schildkraut JM, Halabi S, Bastos E, Marchbanks PA, McDonald JA, Berchuck A (2000) Prognostic factors in early-onset epithelial ovarian cancer: a population-based study. Obstet Gynecol 95: 119–127 10636514 10.1016/s0029-7844(99)00535-9

[bib27] Sevelda P, Vavra N, Schemper M, Salzer H (1990) Prognostic factors for survival in stage I epithelial ovarian carcinoma. Cancer 65: 2349–2352 2346919 10.1002/1097-0142(19900515)65:10<2349::aid-cncr2820651031>3.0.co;2-#

[bib28] Sjovall K, Nilsson B, Einhorn N (1994) Different types of rupture of the tumor capsule and the impact on survival in early ovarian carcinoma. Int J Gynecol Cancer 4: 333–336 11578428 10.1046/j.1525-1438.1994.04050333.x

[bib29] Soper JT, Johnson P, Johnson V, Berchuck A, Clarke-Pearson DL (1992) Comprehensive restaging laparotomy in women with apparent early ovarian carcinoma. Obstet Gynecol 80: 949–953 1333065

[bib30] Surveillance, Epidemiology and End Results (SEER) Program (2005) SEER^*^Stat Database: Incidence – SEER 12 Regs Public-Use, November 2004 Sub (1988–2001), National Cancer Institute, DCCPS, Surveillance Research Program, Cancer Statistics Branch. Available at http://seer.cancer.gov/. Released April 2005

[bib31] Trope C, Kaern J, Hogberg T, Abeler V, Hagen B, Kristensen G, Onsrud M, Pettersen E, Rosenberg P, Sandvei R, Sundfor K, Vergote I (2000) Randomized study on adjuvant chemotherapy in stage I high-risk ovarian cancer with evaluation of DNA-ploidy as prognostic instrument. Ann Oncol 11: 281–288 10811493 10.1023/a:1008399414923

[bib32] Vergote IB, Kaern J, Abeler VM, Pettersen EO, De Vos LN, Trope CG (1993) Analysis of prognostic factors in stage I epithelial ovarian carcinoma: importance of degree of differentiation and deoxyribonucleic acid ploidy in predicting relapse. Am J Obstet Gynecol 169: 40–52 8333474 10.1016/0002-9378(93)90129-7

[bib33] Yancik R (1993) Ovarian cancer. Age contrasts in incidence, histology, disease stage at diagnosis, and mortality. Cancer 71: 517–523 8420671 10.1002/cncr.2820710205

[bib34] Young RC, Decker DG, Wharton JT, Piver MS, Sindelar WF, Edwards BK, Smith JP (1983) Staging laparotomy in early ovarian cancer. JAMA 250: 3072–3076 6358558

[bib35] Young RC, Walton LA, Ellenberg SS, Homesley HD, Wilbanks GD, Decker DG, Miller A, Park R, Major Jr F (1990) Adjuvant therapy in stage I and stage II epithelial ovarian cancer. Results of two prospective randomized trials. N Engl J Med 322: 1021–1027 2181310 10.1056/NEJM199004123221501

